# Estimating influenza and respiratory syncytial virus-associated mortality in Western Kenya using health and demographic surveillance system data, 2007-2013

**DOI:** 10.1371/journal.pone.0180890

**Published:** 2017-07-07

**Authors:** Gideon O. Emukule, Peter Spreeuwenberg, Sandra S. Chaves, Joshua A. Mott, Stefano Tempia, Godfrey Bigogo, Bryan Nyawanda, Amek Nyaguara, Marc-Alain Widdowson, Koos van der Velden, John W. Paget

**Affiliations:** 1 Centers for Disease Control and Prevention - Kenya Country Office, Nairobi, Kenya; 2 Radboud University Medical Center, Department of Primary and Community care, Nijmegen, The Netherlands; 3 Netherlands Institute for Health Services research (NIVEL), Utrecht, The Netherlands; 4 Influenza Division, National Center for Immunization and Respiratory Diseases, US Centers for Disease Control and Prevention, Atlanta, Georgia, United States; 5 US Public Health Service, Rockville, Maryland, United States of America; 6 Center for Respiratory Diseases and Meningitis, National Institute for Communicable Diseases of the National Health Laboratory Service, Johannesburg, South Africa; 7 Kenya Medical Research Institute, Kisumu, Kenya; University of Hong Kong, HONG KONG

## Abstract

**Background:**

Influenza and respiratory syncytial virus (RSV) associated mortality has not been well-established in tropical Africa.

**Methods:**

We used the negative binomial regression method and the rate-difference method (i.e. deaths during low and high influenza/RSV activity months), to estimate excess mortality attributable to influenza and RSV using verbal autopsy data collected through a health and demographic surveillance system in Western Kenya, 2007–2013. Excess mortality rates were calculated for a) all-cause mortality, b) respiratory deaths (including pneumonia), c) HIV-related deaths, and d) pulmonary tuberculosis (TB) related deaths.

**Results:**

Using the negative binomial regression method, the mean annual all-cause excess mortality rate associated with influenza and RSV was 14.1 (95% confidence interval [CI] 0.0–93.3) and 17.1 (95% CI 0.0–111.5) per 100,000 person-years (PY) respectively; and 10.5 (95% CI 0.0–28.5) and 7.3 (95% CI 0.0–27.3) per 100,000 PY for respiratory deaths, respectively. Highest mortality rates associated with influenza were among ≥50 years, particularly among persons with TB (41.6[95% CI 0.0–122.7]); and with RSV were among <5 years. Using the rate-difference method, the excess mortality rate for influenza and RSV was 44.8 (95% CI 36.8–54.4) and 19.7 (95% CI 14.7–26.5) per 100,000 PY, respectively, for all-cause deaths; and 9.6 (95% CI 6.3–14.7) and 6.6 (95% CI 3.9–11.0) per 100,000 PY, respectively, for respiratory deaths.

**Conclusions:**

Our study shows a substantial excess mortality associated with influenza and RSV in Western Kenya, especially among children <5 years and older persons with TB, supporting recommendations for influenza vaccination and efforts to develop RSV vaccines.

## Introduction

Influenza viruses and respiratory syncytial virus (RSV) cause substantial morbidity globally [1–5]. Disease severity associated with influenza and RSV has been well described in temperate countries as pronounced among young children [3, 6, 7], older persons [3, 7, 8], and among persons with chronic medical conditions [4–6, 9]. In Kenya, influenza virus and RSV circulate year round and although limited morbidity data exist [10–15], associated mortality has not been established. Influenza and RSV associated mortality can inform policy makers in low and middle income countries to prioritize segments of the population that are most in need of the potentially limited vaccination and treatment resources.

Conceptually, excess mortality can be estimated as the difference between observed mortality (during the periods of influenza or RSV circulation) and expected mortality (if the pathogens are not circulating) [16–20]. In Kenya, as in most sub-Saharan African countries, there is an absence of systematically collected and robust vital statistics data. Furthermore, hospital-based data could under-represent the number of those who die from respiratory illness as care-seeking is low, particularly among adults who are often underrepresented in health-facility based surveillance systems [21, 22].

Here, we used verbal autopsy (VA) data collected through a health and demographic surveillance system (HDSS) in Western Kenya [23–25] to estimate the overall and age-specific excess mortality rates associated with influenza virus and RSV during the period 2007 to 2013. We also explore and discuss the merits and challenges of using two different estimation methods: (i) the negative binomial regression method which has been used in temperate countries to estimate excess mortality associated with influenza and RSV [16–19], and (ii) the rate-difference method which has previously been recommended as the method of choice when estimating excess mortality for countries without a clear disease seasonality pattern [16, 20].

## Methods

### Study site and population

The Western Kenya HDSS has been in existence since 2001 and currently comprises three geographical sites in Nyanza Province: Asembo, Gem and Karemo, [23, 25]. These three areas cover an estimated area of 700 km^2^ with a culturally homogenous rural population of approximately 220,000 [23]. Nyanza Province also has a high burden of malaria, pulmonary tuberculosis (TB) [26] and human immunodeficiency virus (HIV) which has prevalence of 15% compared to 6% overall in Kenya [27]. Currently, there is no publicly funded influenza vaccination program in Kenya and a limited number of doses are available and distributed annually (approximately 30, 0000 doses), mostly through the private health sector [11].

### Mortality and population data sources

Our study-participants included HDSS residents, i.e., lived in the site for at least four consecutive months, and their infants [23]. Participants live in compounds (or households) that are geo-spatially mapped, and each person receives a unique identification number allowing record linkage. A household census is conducted throughout the study area every four months to capture births, pregnancies, deaths, in- and out- migration, and economic data [23, 28]. If a death is reported, at least one month after the death trained interviewers use a standardized World Health Organization (WHO) questionnaire [29] endorsed by International Network for the Demographic Evaluation of Populations and Their Health (INDEPTH) to collect information on the decedent’s illness and care seeking behavior [30]. The cause of death is assigned using the InterVA-4 method with corresponding ICD-10 codes (S1 File).

### Influenza, RSV and malaria activity and data sources

Indices of influenza, RSV and malaria activity were defined using the monthly positive proportion of tested samples from our hospital-based active surveillance sites. These sites serve the same population that includes the decedents for whom VA data were collected. Malaria was considered *a priori* as a potential confounder because it is endemic in the study region and has demonstrated seasonal mortality that sometimes overlaps with influenza and RSV activity. Malaria test result, as well as influenza and RSV virological data, were collected from three HDSS surveillance sites [Siaya County Referral Hospital (SCRH), Lwak Mission Hospital (Lwak), and Ting’wang’i Health Center (THC)]. Patients at these facilities were screened for malaria using methods previously described [31]. Nasopharyngeal (NP) and oropharyngeal (OP) swabs were collected from patients (of all ages) with influenza-like illness (ILI) who presented as outpatients at Lwak or at THC, or patients hospitalized with severe acute respiratory illness (SARI) at Lwak or SCRH. (See S1 File for case definitions). Laboratory testing for influenza A and B viruses and RSV was performed by real-time reverse transcription polymerase chain reaction (rRT-PCR) using CDC protocols [32, 33].

### Data analyses

Estimates of deaths associated with influenza and RSV were calculated for four mortality outcomes: (i) all-cause deaths, (ii) respiratory deaths (including pneumonia), (iii) HIV-related deaths, and (iv) TB related deaths. For each decedent case, the cause of death was assigned as respiratory, HIV-, and TB-related if it was listed as a probable cause of death. We did not estimate excess mortality for circulatory deaths associated with influenza and RSV because of the relatively low number of deaths recorded.

#### Descriptive analyses and handling of missing VA data

The demographic characteristics of those who died were described using medians and ranges. Wilcoxon rank-sum and Chi-square tests were used to assess if there were differences between the age and sex distributions of cases with VA-coded underlying cause of death and those without. Influenza and RSV circulation patterns were described using monthly percentages of positive results. We adjusted for missing VA data (age groups: <5, 5–49, ≥50 years, all ages), by dividing the monthly number of outcome-specific (all-causes, all-respiratory, HIV-, and TB-related) deaths by the monthly proportion of deaths with VA done.

#### Estimating excess deaths using the negative binomial regression method

Negative-binomial regression models which incorporated monthly influenza and RSV circulation data and adjusted for malaria activity were used to estimate the age-specific and pathogen-associated deaths. For each age group and mortality outcome, we explored a range of models with varying combinations of time polynomial terms (up to the 6^th^ order) and seasonal cyclical terms (starting with a full model that incorporated time polynomials, the quarterly, semiannual and annual seasonal cyclical terms). The general model equation was of the form:

$$\begin{array}{l} E\left(Y_{t}\right)=\beta_{0}+\beta_{1}(t)+\beta_{2}(t_{}^{2})+\ldots +\beta_{6}(t_{}^{6})+\beta_{7}\left[\text{sin}\left(2t\pi /12\right)\right]+\beta_{8}\left[\text{cos}\left(2t\pi /12\right)\right]+\beta_{9}\left[\text{sin}\left(2t\pi /6\right)\right]\\ +\beta_{10}\left[\text{cos}\left(2t\pi /6\right)\right]+\beta_{11}\left[\text{sin}\left(2t\pi /3\right)\right]+\beta_{12}\left[\text{cos}\left(2t\pi /3\right)\right]+\beta_{13}\left[Influenza\right]\\ +\beta_{14}\left[RSV\right]+\beta_{15}\left[Malaria\right]+\varepsilon_{t} \end{array}$$

Where *E(Y*_*t*_*)* is the age-specific number of deaths; *t* is the running time variable; *β*_*0*_ is the model constant; *β*_*1*_ to *β*_*6*_ are the coefficients for the linear and polynomial time trends; *β*_*7*_ to *β*_*12*_ are the coefficients corresponding to the cyclical terms for the annual, semiannual and quarterly background seasonal variations; *β*_*13*_, *β*_*14*_ and *β*_*15*_ are the coefficients representing the contribution of influenza, RSV and malaria respectively, and *ε*_*t*_ is the error term. The final model that was selected was the one for which the Akaike Information Criterion (AIC) values were minimized.

We explored using the natural cubic spline smoothing functions of time to model the background mortality as opposed to using the polynomial time trends and sinusoidal curves but the estimates were not significantly different. However, as our time series data were monthly and only analyzed over a period of seven years, we were concerned about overfitting the models when we used the spline method. Therefore, the mortality estimates that we report here are based on models that incorporated sinusoidal curves to model the background mortality.

To estimate the excess mortality associated with a specific pathogen (influenza or RSV), we first calculated the predicted monthly deaths from the model that included a term for the detection of the pathogen (full model) and then subtracted the predicted deaths from the baseline model (where the term for the detection of the pathogen was set to zero) [6, 16, 19, 34]. Only positive differences between the full and the baseline model were considered. We then calculated the age-specific excess mortality rates per 100,000 person-years (PY) by dividing the average annual number of excess deaths by the population at risk. The 95% confidence intervals (CI) were estimated using bootstrap sampling (with replacement) on blocks of calendar years over 1,000 replications [34]. To make the data comparable with those used in the analysis with the rate-difference method, diarrheal deaths were excluded from the all-cause deaths.

#### Estimating excess deaths using the baseline rate-difference method

**Fig 1:**
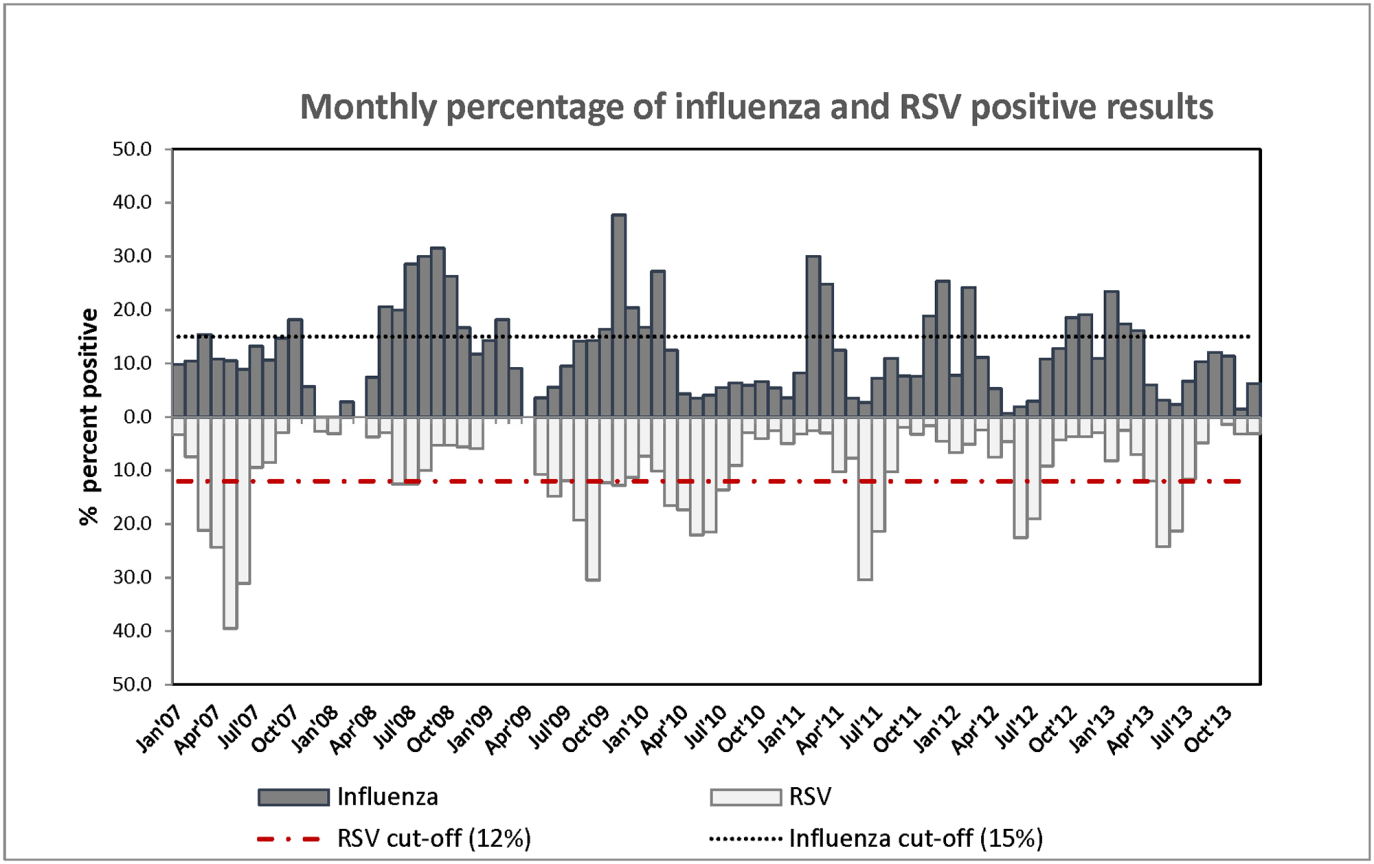
Monthly percentage of influenza and respiratory syncytial virus (RSV) positive results with cut-offs for the rate-difference method.

In this approach, we calculated the excess deaths attributable to influenza and RSV by taking the positive difference between age-specific deaths occurring each month when there was high pathogen circulation and the monthly average of deaths that occurred during the months of low pathogen circulation (baseline months) [16, 20]. Months when the percentage of influenza and RSV cases were less than 15% and 12%, respectively, were considered as the baseline months (Fig 1). These baselines were defined based on the upper limit of the 95% CI on the mean pathogen detection rate over the study period (S1 File). To avoid double counting deaths for months where both influenza and RSV activity exceed these thresholds, we apportioned excess deaths for each pathogen proportionate to how the pathogen activity deviated from the stated thresholds (15% and 12%) (S1 File). Excess mortality rates were calculated as the mean annual number of excess deaths divided by the age-specific population at risk. The Poisson approximation method was used to calculate the 95% CI around point estimates [35]. In this analysis, malaria and diarrheal deaths were excluded from the all-cause deaths as they tended to follow a seasonal pattern similar to the RSV activity pattern (S1 Figs).

## Ethical considerations

The HDSS protocol and consent procedures, were approved by the Ethical Review Committee of the Kenya Medical Research Institute (KEMRI SSC-1801) and the Institutional Review Board of CDC-Atlanta (CDC IRB #3308).

## Results

### Descriptive analyses

From January 2007 through December 2013, the Western Kenya HDSS population increased by 14% from 218,985 to 249,470. Over this period, there were a total of 22,899 deaths reported. Of these, 19,991 (87%) had VA conducted and a cause of death assigned. The median age of death was 32 years [interquartile range (IQR) 2–63 years]. There were no differences between those with a VA-coded cause of death and those without by sex (χ = 0.8692, p = 0.351). However, those without a VA-coded cause of death were younger [median age 27 years (IQR = 2–46) vs. 33 years (IQR = 2–65); p<0.001]. The proportion of deaths without VA data also varied by year and was highest in 2007 (17%) and lowest in 2012 (8%).

A total of 13,677 and 10,001 samples were collected and tested for influenza and RSV, respectively, over the study period. Of these, 1,620 (12%) tested positive for influenza viruses and 1,022 (10%) tested positive for RSV. The average monthly percentage of tested patients who were positive for influenza was 12% (95% CI 10–14) and for RSV was 9% (95% CI 7–11) (Fig 1, and S1 Figs). Malaria parasites were detected in 43% of all patients evaluated over the entire study period.

### Overall mortality rates and trends

**Fig 2:**
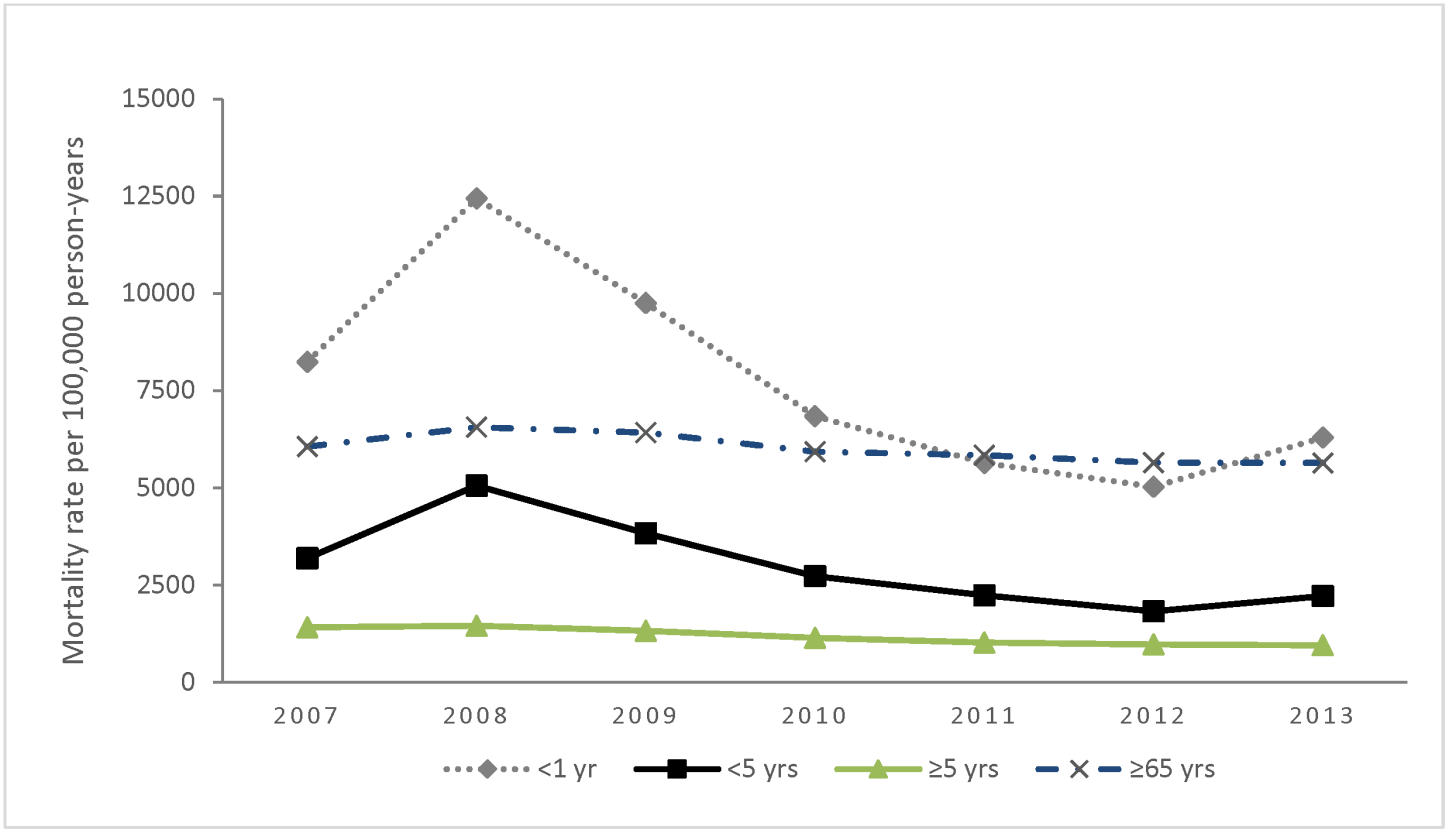
Age-specific trends in all-causes mortality rates in Western Kenya, 2007–2013.

Over the study period, the mean annual all-causes mortality rate was 1,446 (95% CI 1,397–1,497)/100,000 PY. Among children <5 years the annual all-causes mortality rate ranged from 1,827 to 5,059 deaths per 100,000 PY (lowest in 2012 and highest in the 2008), for a mean of 2,965 (95% CI 2,791–3,150)/100,000 (Table 1 and Fig 2). Among persons aged ≥5 years, the annual all-causes mortality rate ranged from 953 to 1,456 per 100,000 PY for a mean of 1,163 (95% CI 1,115–1,212)/100,000 PY. The mean annual respiratory mortality rate was 148 (95% CI 133–164)/100,000 PY, and among children <5 years was 513 (95% CI 443–593)/100,000 PY]. The mean annual mortality rates for HIV- and TB-related deaths were 275 (95% CI 254–298) and 186 (95% CI 169–204) per 100,000 PY, respectively.

**Table 1:** Age-specific mean annual mortality rates in Western Kenya, January 2007—December 2013.

Age group	Person-years	All-cause mortality* (95% CI)	Respiratory mortality (including pneumonia)* (95% CI)	HIV-related mortality* (95% CI)	Pulmonary TB (TB) related mortality* (95% CI)
0–11 months	6,956	7,690 (7,065–8,370)	1,896 (1,598–2,249)	704 (532–932)	0 (-)
12–23 months	7,059	3,908 (3,473–4,397)	403 (279–582)	1,139 (916–1,418)	18 (3–103)
24–59 months	21,271	1,107 (975–1,258)	97 (63–150)	203 (151–274)	6 (1–34)
<5 years	35,286	2,965 (2,791–3,150)	513 (443–593)	489 (422–568)	7 (2–25)
5–14 years	65,209	213 (181–252)	21 (13–36)	39 (26–57)	8 (3–19)
15–49 years	93,149	1,025 (962–1,092)	54 (41–71)	340 (304–379)	254 (223–288)
50–64 years	17,847	1,923 (1,730–2,137)	138 (93–204)	395 (313–499)	364 (286–464)
≥65 years	12,737	5,964 (5,554–6,403)	482 (376–619)	253 (180–358)	840 (695–1,015)
≥50 years	30,584	3,606 (3,399–3,825)	281 (228–347)	336 (277–408)	562 (484–653)
≥5 years	188,943	1,163 (1,115–1,212)	79 (68–93)	235 (214–258)	219 (199–241)
Total	224,228	1,446 (1,397–1,497)	148 (133–164)	275 (254–298)	186 (169–204)

*Deaths per 100,000 person-years;

CI-Confidence intervals; HIV-Human immunodeficiency virus

### Influenza- and RSV-associated deaths using the negative binomial regression method

**Fig 3:**
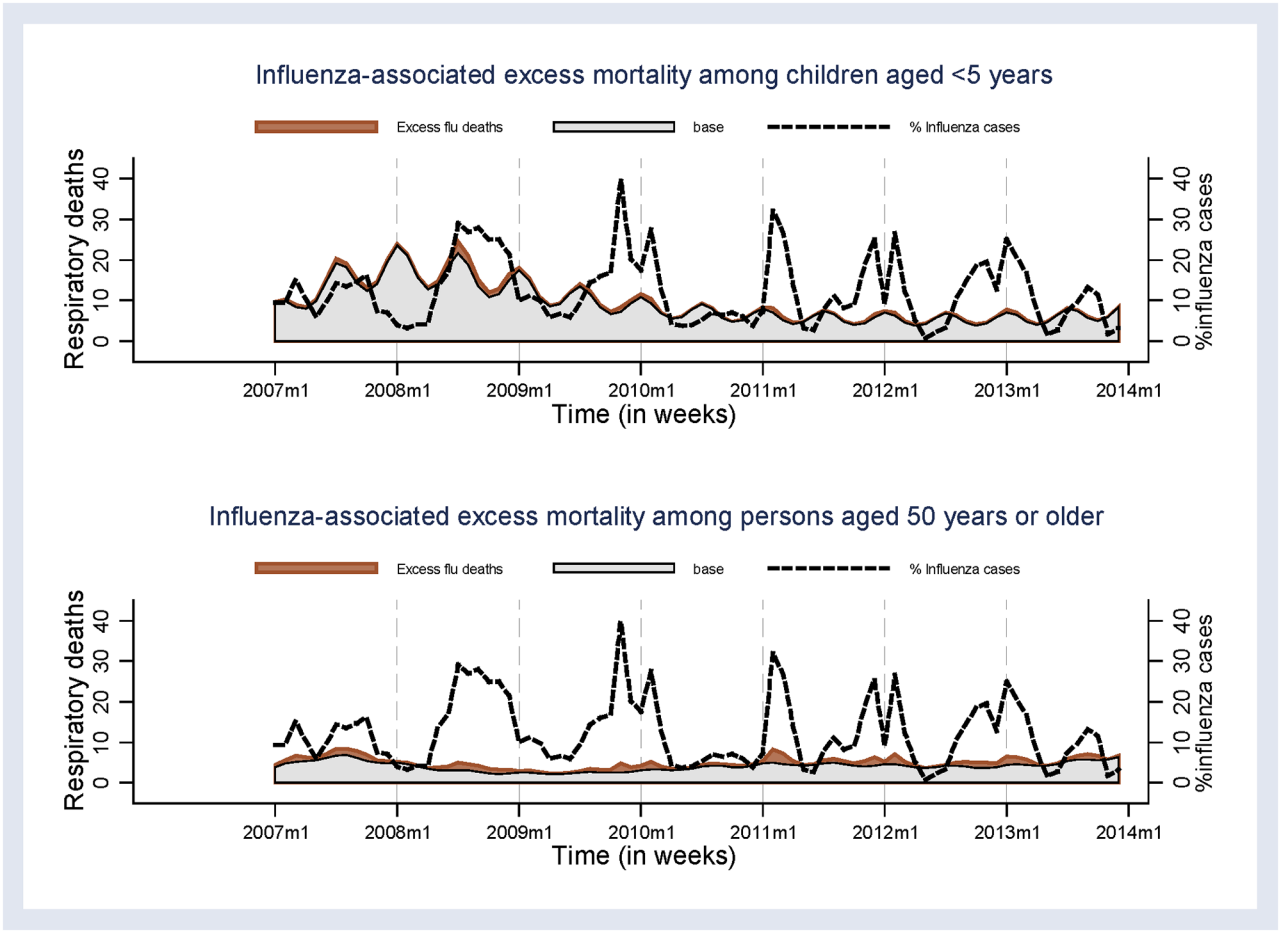
Excess deaths estimated using the negative binomial regression model that were associated with influenza among children aged <5 years and persons aged ≥50 years, 2007–2013.

Using the negative binomial regression method, the mean annual excess all-causes mortality rate associated with influenza activity was 14.1 (95% CI 0.0–93.3)/100,000 PY; and was highest among persons aged ≥50 years (74.0 [95% CI 0.0–310.4]/100,000 PY) (Table 2 and Fig 3). Similarly, the mean annual excess mortality rate for respiratory deaths was highest among persons aged ≥50 years (34.6 [95% CI 0.0–81.0]/100,000 PY). Among children aged <5 years, the mean annual excess mortality rate of respiratory deaths associated with influenza was 16.7 (95% CI 0.0–78.2)/100,000 PY. The mean annual excess TB related mortality rate was highest among persons aged ≥50 years (41.6 [95%CI 0.0–122.7]/100,000 PY).

**Table 2:** Age-specific mean annual excess mortality rate associated with influenza in Western Kenya, 2007–2013.

Cause of death by age	Negative-binomial regression method		Rate-difference method (High^a^ activity vs. baseline^b^)	
	Estimated deaths	Mortality Rate* (95% CI)	Estimated deaths	Mortality Rate* (95% CI)
All causes				
<5 years	8	22.2 (0.0–145.2)	44	125.5 (93.4–168.4)
5–49 years	1	0.8 (0.0–40.0)	37	23.6 (17.1–32.5)
≥50 years	23	74.0 (0.0–310.4)	29	95.1 (66.1–136.7)
All ages	32	14.1 (0.0–93.3)	100	44.8 (36.8–54.4)
Respiratory, including pneumonia				
<5 years	6	16.7 (0.0–78.2)	22	62.7 (41.3–95.1)
5–49 years	7	4.5 (0.0–7.2)	4	2.2 (0.8–6.3)
≥50 years	11	34.6 (0.0–81.0)	5	17.7 (7.6–41.1)
All ages	24	10.5 (0.0–28.5)	22	9.6 (6.3–14.7)
HIV/AIDS				
<5 years	1	3.6 (0.0–27.3)	14	40.9 (24.4–68.6)
5–49 years	NE	NE	9	6.3 (3.4–11.7)
≥50 years	NE	NE	4	13.4 (5.1–35.2)
All ages	NE	NE	25	11.3 (7.7–16.7)
Pulmonary Tuberculosis (TB)				
<5 years	1	2.2 (0.0–8.2)	1	2.8 (0.4–20.1)
5–49 years	10	6.5 (0.0–31.6)	27	17.4 (11.9–25.2)
≥50 years	13	41.6 (0.0–122.7)	13	41.8 (24.1–72.3)
All ages	24	10.6 (0.0–40.2)	39	17.5 (12.8–23.9)

^a^Monthly percentage of influenza positive cases ≥15%;

^b^Monthly percentage of influenza cases <15%;

*Deaths per 100,000 person-years.

NE-Not estimated

**Fig 4:**
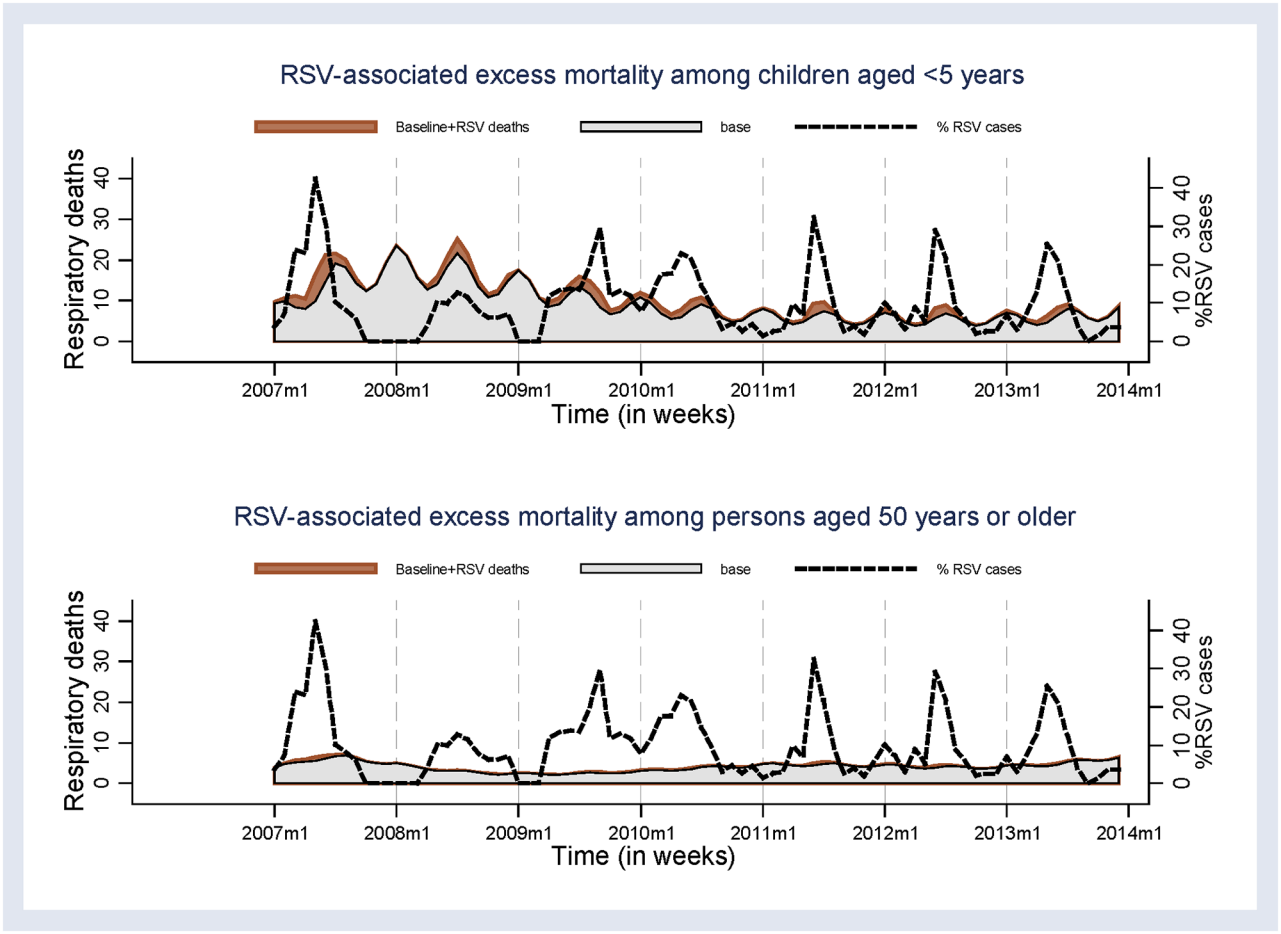
Excess deaths estimated using the negative binomial regression model that were associated with RSV among children aged <5 years and persons aged ≥50 years, 2007–2013.

The mean annual excess all-causes mortality rate associated with RSV was 17.1 (95% CI 0.0–111.5)/100,000 PY. In contrast to influenza, the mean annual excess RSV mortality rate for respiratory deaths was highest among children aged <5 years (38.5 [95% CI 0.0–109.9]/100,000 PY) (Table 3 and Fig 4).

**Table 3:** Age-specific mean annual excess mortality rate associated with respiratory syncytial virus (RSV) in Western Kenya, 2007–2013.

Cause of death by age	Negative-binomial regression method		Rate-difference method (High^a^ activity vs. baseline^b^)	
	Estimated deaths	Mortality Rate* (95% CI)	Estimated deaths	Mortality Rate* (95% CI)
All causes				
<5 years	12	32.6 (0.0–397.1)	32	90.3 (63.8–127.8)
5–49 years	6	3.7 (0.0–28.7)	9	5.5 (2.8–10.6)
≥50 years	21	68.7 (0.0–208.0)	15	48.2 (28.9–80.3)
All ages	39	17.1 (0.0–111.5)	44	19.7 (14.7–26.5)
Respiratory, including pneumonia				
<5 years	14	38.5 (0.0–109.9)	13	38.1 (22.3–65.0)
5–49 years	1	0.6 (0.0–8.4)	2	1.0 (0.2–4.8)
≥50 years	2	5.9 (0.0–29.0)	1	4.7 (0.9–24.1)
All ages	17	7.3 (0.0–27.3)	15	6.6 (3.9–11.0)
HIV/AIDS				
<5 years	1	3.7 (0.0–87.0)	19	53.7 (34.2–84.2)
5–49 years	NE	NE	11	6.7 (3.6–12.2)
≥50 years	NE	NE	3	10.9 (3.7–31.9)
All ages	NE	NE	29	13.1 (9.1–18.8)
Pulmonary Tuberculosis (TB)				
<5 years	1	1.8 (0.0–160.3)	1	2.1 (0.2–20.5)
5–49 years	2	1.4 (0.0–31.4)	7	4.3 (2.0–9.1)
≥50 years	11	35.1 (0.0–73.4)	9	28.3 (14.5–55.1)
All ages	14	6.0 (0.057.6)	12	5.2 (2.9–9.2)

^a^Monthly percentage of RSV positive cases ≥12%;

^b^Monthly percentage of RSV cases <12%;

*Deaths per 100,000 person-years.

NE-Not estimated

### Excess deaths using the rate-difference method

The mean annual excess all-causes mortality rate associated with influenza that was estimated using the rate difference method was 44.8 (95% CI 36.8–54.4)/100,000 PY. Respiratory influenza-associated mortality among <5 years was 62.7 (95% CI 41.3–95.1) compared to 17.7 (95% CI 7.6–41.1)/100,000 PY among ≥50 year olds. Of HIV-related deaths, the mean annual excess influenza-associated mortality rate was highest among <5 years (40.9 [95% CI 24.4–68.6] /100,000 PY), whereas TB related mortality rate associated with influenza was highest among persons aged ≥50 years (41.8 [95% CI24.1–72.3] /100,000 PY) (Table 2).

The mean annual excess all-causes mortality rate associated with RSV was highest among <5 years (90.3 [95% CI 63.8–127.8]/100,000 PY) (Table 3). Rate estimates of respiratory deaths associated with RSV was highest among children <5 years (38.1 [95% CI 22.3–65.0]/ 100,000 PY), whereas among persons aged ≥50 years was 4.7 (95% CI 0.9–24.1) /100,000 PY).

## Discussion

Using two different methods (negative binomial regression and the rate-difference), our study showed that both influenza and RSV are associated with mortality among children <5 years, and adults ≥50 years. The two estimation methods were consistent, for most of the mortality outcomes and age groups considered, in showing the similar segments of the population with high mortality burden.

The rate-difference estimation approach has been suggested for estimating excess deaths where there are multiple peaks with no clear seasonality patterns, or where there is only a limited time series of data available (less than five years of data) [16]. However, its major drawback is its inability to account for the effect of other pathogens that co-circulate with the pathogen investigated. In a region like Western Kenya where malaria is endemic [13], the rate-difference method could potentially overestimate influenza-associated excess deaths. Moreover, as both influenza and RSV circulated throughout the year, for most of the data included in the analysis, it was computationally difficult to disentangle the possible effect of one pathogen on estimates of the other using the rate-difference method. Additionally, incidences where both pathogens co-circulate at levels that exceed the specified thresholds could potentially lead to double counting excess deaths using the rate-difference method. In our analysis, we attempted to correct for this by apportioning excess deaths based on the degree by which the pathogen activity, as measured by the proportion of those who tested positive, deviated from the expected (S1 File). However, the limitation of this approach is that it assumes that the case-fatality rates for influenza and RSV are similar, which may not necessarily be the case.

Unlike the case with the rate-difference method, the negative binomial regression models were adjusted for the effect of RSV/influenza and malaria. This could partially explain the consistently lower estimates using this method when compared to estimates obtained using the rate-difference method. Notwithstanding its analytical advantage over the rate-difference method, we believe that the data that we used in our analysis, which were collected from a HDSS population of about 220,000 in Western Kenya, were underpowered to estimate excess mortality using regression methods. Indeed, this could explain why we did not observe statistically significant excess mortality estimates associated with influenza and RSV as we had very wide confidence intervals which also overlapped with zero. This supports the need for future analyses when more robust, and nationally representative data become available. More robust analysis could be used to estimate multipliers for the point estimates derived from the binomial regression methods relative to the rate-difference methods. Such multipliers, if consistent, could be used by countries with similar settings as Kenya but with limited data to adjust for the inadequacies of the rate-difference method when estimating excess mortality rates.

It is not clear which death outcome category is more appropriate to characterize excess mortality associated with influenza or RSV in countries where these diseases have no marked seasonality and where there are competing causes of deaths such as malaria, TB, and HIV. As in other studies [8, 17, 36, 37], our estimates for all-causes excess mortality were higher than estimates for respiratory excess mortality for both influenza and RSV due to its low specificity. In appreciation of this challenge, most of studies try to make estimates available using several death outcomes including pneumonia and influenza and cardiovascular disease [7, 8, 17]; however, the underlying characteristics of the population in sub-Saharan Africa may differ from those in temperate countries which may limit the utility of this approach.

All-causes mortality rates varied across the period of analysis in our study with the highest mortality rates observed in the year 2008. In that year, mortality rates were particularly higher among children aged under five years, and were nearly three times higher than the in the year 2012 when the mortality rate was lowest. One of the possible explanations to the observed increase in mortality rates among children aged under five years was as a result of the civil unrest associated with post-election violence in Kenya that began in December, 2007 and resulted in the disruption of health care services and straining of household resources [38].

Our estimates of all-causes excess mortality associated with influenza are comparable to estimates from Singapore [39], Hong Kong [37, 40, 41], and New Zealand [42] among persons of all ages. Estimates of respiratory excess deaths associated with influenza among children <5 years were comparable to the estimates reported in South Africa [6], but over ten-fold higher than rates reported in the United States [7, 43]. The TB related excess mortality that was associated with influenza was higher among persons ≥50 years compared to children, consistent with other studies [34], highlighting the risk for influenza-associated complications among TB patients and the potential impact of influenza vaccination in areas of high prevalence of TB. Because of the relatively low number of deaths, we were not able to estimate the excess HIV-related deaths that were associated with influenza for various age groups using the negative binomial regression method. However, estimates from the rate-difference method suggest an important HIV-related mortality associated with influenza among children aged <5 years. Further analysis may be warranted when more data become available as studies conducted in South Africa have suggested a higher mortality rate associated with influenza among persons with HIV compared to those without [6, 8].

Although the confidence intervals suggest comparable respiratory mortality rates associated with influenza and RSV among children aged <5 years, using the negative binomial regression method, our study found relatively higher point estimates for mortality rates in this age group associated with RSV compared to influenza, which is consistent with findings from studies conducted elsewhere [7, 43]. The estimates of excess mortality associated with RSV were higher than rates reported in South Africa [6], and in the United States [7, 43]. We also noted a high all-cause mortality associated with RSV among persons aged ≥50 years, which was similar to that associated with influenza and consistent with other studies that have suggested RSV as an important cause of morbidity and mortality among older adults [43, 44].

Other than the data limitations discussed earlier, our study was subject to other important limitations. First, we used VA data and not clinician certified cause-of-death data and thus estimates may vary as a function of the true cause of death composition in the population. Indeed, a study conducted elsewhere estimated the cause-specific mortality fraction accuracy at 0.625 and 0.629 for adults and children respectively, using verbal autopsy with the cause of death assigned using the InterVA-4 method [45]. Second, the data used in our analysis were collected from Western Kenya and may not be representative of the overall Kenyan population. Lastly, we could not evaluate the impact of different virus subtypes on excess mortality due to limited data available.

In conclusion, despite the data limitations, our study suggests a role of influenza and RSV on excess mortality in Western Kenya, especially among children <5 years and persons ≥50 years. These data suggest that future RSV vaccines [46, 47], and vaccination of children, older adults and persons with chronic medical conditions against seasonal influenza has the potential to reduce mortality rates in Western Kenya.

## Supporting information

S1 File Supplementary methods. (DOCX)

S1 Figs Supplementary figures. (DOCX)
