# Supplementary material for: Estimating influenza and respiratory syncytial virus-associated mortality in Western Kenya using health and demographic surveillance system data, 2007-2013
Source: PLoS One. 2017 Jul 7;12(7):e0180890. doi: 10.1371/journal.pone.0180890 (PMC5501643; doi:10.1371/journal.pone.0180890)
Supplement: S1 File — (DOCX) [file pone.0180890.s001.docx]

**Supplemental File 1. Methods**

**Case definitions**

*Influenza-Like Illness*

For patients of all ages, influenza-like illness was defined as an axillary temperature ≥38^o^C and cough or sore throat in an outpatient [[1](#_ENREF_1), [2](#_ENREF_2)].

*Severe Acute Respiratory Illness (SARI)*

SARI was defined differently for children under five years and for persons aged ≥5 years. Among children aged <5 years, SARI was defined using a modified version of the World Health Organization’s Integrated Management of Childhood Illness (IMCI) definition for pneumonia. This was defined as hospitalization with cough OR difficulty breathing, AND at least one of (maternal report of lower-chest wall in-drawing, stridor in a calm child, unable to drink or breast feed, vomiting, convulsions, lethargic or unconscious, oxygen saturation <90%). Among persons aged 5 years, SARI was defined as hospitalization with cough OR difficulty breathing OR shortness of breath AND a documented fever (≥38^o^C) [[1](#_ENREF_1), [2](#_ENREF_2)]. At Lwak Mission hospital, a modified “SARI” case definition that did not require hospitalization was used. Among children <5 years, SARI was defined as defined as cough OR difficulty breathing, AND at least one of (inability to drink or breastfeed, vomiting everything, convulsions, lethargy or unconscious, lower-chest wall in-drawing, stridor, oxygen saturation <90%). For persons aged ≥5 years, SARI was defined as cough OR difficulty breathing OR chest pain, AND a documented axillary temperature of ≥38.0°C OR and oxygen saturation level of ≤90% [[1](#_ENREF_1)].

**Verbal autopsy data**

The InterVA-4 model software which was introduced in the year 2009 was used to assign the possible cause of death. To maintain uniformity, data that were collected prior to its introduction were re-assigned a cause of death using the InterVA-4 software. It is important to mention that data from this HDSS area were included in a multi-country study that compared the physician–coded verbal autopsy (PCVA) cause of death to the cause of death as determined by the InterVA-4 model [[3](#_ENREF_3)]. Results from this analysis showed a very strong correlation 0.83 (95% CI 0.75 to 0.91) among the five participating countries. Specifically for the Western Kenya HDSS data (with 21,236 deaths), the correlation was 0.85 (95% CI 0.79 - 0.92) [[3](#_ENREF_3)]. Table 1 below shows the cause-of-death list for the mortality outcomes that we considered with the corresponding ICD-10 codes[[4](#_ENREF_4)].

Table 1: Cause-of-death list for verbal autopsy with corresponding broad ICD-10 codes

| **Verbal autopsy title** | **ICD code** | **ICD title** |
| --- | --- | --- |
| Tuberculosis (TB) | A159 | Respiratory tuberculosis, unspecified, confirmed bacteriologically and histologically |
|  | A169 | Respiratory tuberculosis, unspecified, without mention of bacteriological or histological confirmation |
|  | A179 | Tuberculosis of nervous system, unspecified |
|  | A192 | Acute miliary tuberculosis, unspecified |
|  | A199 | Miliary tuberculosis, unspecified |
| HIV/AIDS | B209 | HIV disease resulting in unspecified infectious or parasitic disease |
|  | B219 | HIV disease resulting in unspecified malignant neoplasm |
|  | B227 | Human immunodeficiency virus [HIV] disease resulting in multiple diseases classified elsewhere |
|  | B238 | Human immunodeficiency virus [HIV] disease resulting in other specified conditions |
|  | B24 | Unspecified human immunodeficiency virus [HIV] disease |
| Acute lower respiratory infections (including pneumonia and acute bronchitis) | J129 | Viral pneumonia, unspecified |
|  | J159 | Bacterial pneumonia, unspecified |
|  | J180 | Bronchopneumonia, unspecified |
|  | J181 | Lobar pneumonia, unspecified |
|  | J182 | Hypostatic pneumonia, unspecified |
|  | J189 | Pneumonia, unspecified |

**Defining influenza and RSV ‘baseline’ and ‘activity months’**

Contrary to temperate countries which are characterized by distinct seasons [[5](#_ENREF_5), [6](#_ENREF_6)], influenza and RSV are detected in Kenya almost throughout the year [[1](#_ENREF_1), [2](#_ENREF_2), [7](#_ENREF_7)] without distinct periods of activity, which is particularly true for influenza. We therefore used a cut-off defined using the average monthly percentage of cases detected to define the ‘baseline’ and ‘increased activity’ months for influenza and RSV that were used for the estimation of excess mortality using the rate-difference method. From the distribution of the monthly percentage of influenza positive cases over the entire study period, we determined that the average was 12% (95% CI 10-14). Months where ≥15% (higher than the upper confidence limit on the average percentage) cases of influenza were detected were classified as increased influenza activity months. Similarly, the average monthly percentage of RSV cases was 9% (95% CI 7-11) and months where ≥12% cases of RSV were detected were classified as increased RSV activity months.

**Dealing with potential double counting of deaths in the rate-difference method**

The following steps were applied in calculating excess deaths associated with influenza and RSV for months when the activity index of both pathogens exceeded the stated thresholds (15% and 12% for influenza and RSV respectively):

1. We standardized the activity index variables (based on the monthly percentage of influenza and RSV cases)
2. The excess deaths were then calculated as the positive difference between the monthly number of deaths and the average monthly number of deaths that occurred when the pathogen circulation was low (<15% and <12% for influenza and RSV respectively).
3. Excess deaths were then apportioned on the basis of pathogen activity, as indicated by the standardized pathogen index as shown below:

$${Excessdeaths}_{flu}=\left( Excessdeaths\times\frac{Z_{flu}}{Z_{flu}+Z_{rsv}} \right)$$

Where:

${Excessdeaths}_{flu}$ = Excess deaths associated with influenza activity for a specific month

$Excessdeaths$ = Excess deaths for a specific month over the average monthly number of deaths that occurred during the months of low influenza activity

$Z_{flu}$ = Standardized index for influenza activity for a specific month

$Z_{rsv}$ = Standardized index for RSV activity for a specific month

**References**

1. Katz MA, Lebo E, Emukule G, Njuguna HN, Aura B, Cosmas L, et al. Epidemiology, seasonality, and burden of influenza and influenza-like illness in urban and rural Kenya, 2007-2010. The Journal of infectious diseases. 2012;206 Suppl 1:S53-60. doi: 10.1093/infdis/jis530. PubMed PMID: 23169973.

2. Emukule GO, Khagayi S, McMorrow ML, Ochola R, Otieno N, Widdowson MA, et al. The burden of influenza and RSV among inpatients and outpatients in rural western Kenya, 2009-2012. PloS one. 2014;9(8):e105543. doi: 10.1371/journal.pone.0105543. PubMed PMID: 25133576; PubMed Central PMCID: PMC4136876.

3. Byass P, Herbst K, Fottrell E, Ali MM, Odhiambo F, Amek N, et al. Comparing verbal autopsy cause of death findings as determined by physician coding and probabilistic modelling: a public health analysis of 54 000 deaths in Africa and Asia. Journal of global health. 2015;5(1):010402. doi: 10.7189/jogh.05.010402. PubMed PMID: 25734004; PubMed Central PMCID: PMC4337147.

4. World Health Organization (WHO). Verbal Autopsy Standards. Available at: <http://apps.who.int/iris/bitstream/10665/43764/1/9789241547215_eng.pdf>. Accessed Mar 8th 2016.

5. Finkelman BS, Viboud C, Koelle K, Ferrari MJ, Bharti N, Grenfell BT. Global patterns in seasonal activity of influenza A/H3N2, A/H1N1, and B from 1997 to 2005: viral coexistence and latitudinal gradients. PloS one. 2007;2(12):e1296. doi: 10.1371/journal.pone.0001296. PubMed PMID: 18074020; PubMed Central PMCID: PMC2117904.

6. Viboud C, Boelle PY, Pakdaman K, Carrat F, Valleron AJ, Flahault A. Influenza epidemics in the United States, France, and Australia, 1972-1997. Emerging infectious diseases. 2004;10(1):32-9. doi: 10.3201/eid1001.020705. PubMed PMID: 15078594; PubMed Central PMCID: PMC3322745.

7. Katz MA, Muthoka P, Emukule GO, Kalani R, Njuguna H, Waiboci LW, et al. Results from the first six years of national sentinel surveillance for influenza in Kenya, July 2007-June 2013. PloS one. 2014;9(6):e98615. doi: 10.1371/journal.pone.0098615. PubMed PMID: 24955962; PubMed Central PMCID: PMC4067481.
