# Supplementary material for: Estimating influenza and respiratory syncytial virus-associated mortality in Western Kenya using health and demographic surveillance system data, 2007-2013
Source: PLoS One. 2017 Jul 7;12(7):e0180890. doi: 10.1371/journal.pone.0180890 (PMC5501643; doi:10.1371/journal.pone.0180890)
Supplement: S1 Figs — (DOCX) [file pone.0180890.s002.docx]

**S1 Figures: Supplemental figures**

**S1 Figure 1:** Influenza, RSV and malaria activity patterns in Western Kenya, 2007-2013

**S1 Figure 2:** Graph showing the observed all-respiratory deaths, predicted deaths, and baseline for children aged <5 years, 2007–2013

**S1 Figure 3:** Graph showing the observed all-respiratory deaths, predicted deaths, and baseline for persons aged 5-49 years, 2007-2013

**S1 Figure 4:** Graph showing the observed all-respiratory deaths, predicted deaths, and baseline for persons aged ≥50 years, 2007-2013

**S1 Figure 5:** Graph showing the observed all-respiratory deaths, predicted deaths, and baseline for persons of all ages, 2007-2013
